# Evaluating the Diagnostic Accuracy of Rapid Malaria Tests in Pediatric Patients: A Retrospective Cohort Study

**DOI:** 10.7759/cureus.70817

**Published:** 2024-10-04

**Authors:** Mohammad Irshad, Inayatullah Khan, Ayesha LNU, Gulmina Shah, Rashida Sadiq, Fahad R Khan

**Affiliations:** 1 Pediatrics, Lady Reading Hospital Medical Teaching Institution, Peshawar, PAK; 2 Pediatric Medicine, Lady Reading Hospital Medical Teaching Institution, Peshawar, PAK; 3 Pediatrics, Peshawar Institute of Medical Sciences, Peshawar, PAK; 4 Medicine, Police Services Hospital, Peshawar, PAK; 5 Cardiology, Lady Reading Hospital Medical Teaching Institution, Peshawar, PAK

**Keywords:** diagnostic accuracy, malaria, microscopy, parasitic load, pediatric malaria, rapid diagnostic test, sensitivity, specificity

## Abstract

Background

Malaria remains one of the most significant public health challenges, particularly in pediatric populations in developing countries. Rapid diagnostic tests (RDTs) have been widely adopted due to their ease of use and quick results, but their diagnostic accuracy, compared to microscopy, the gold standard, remains a topic of interest. Early and accurate detection of malaria is crucial in reducing complications and mortality in children, who are especially vulnerable to delayed or inaccurate diagnoses.

Objective

This study aims to evaluate the diagnostic accuracy of RDTs for diagnosing *Plasmodium falciparum* malaria in children, using microscopy as the gold standard for comparison.

Methods

A retrospective cohort study was conducted in the Pediatric Department of Lady Reading Hospital Medical Teaching Institution (MTI), Peshawar, from January 1, 2023, to December 31, 2023. A total of 140 pediatric patients (aged 12 years or below) who were clinically suspected of malaria were included. Patients were excluded if they had a prior confirmed diagnosis of malaria before admission, were already receiving anti-malarial treatment, or if their guardians did not provide consent for participation. Blood samples were collected from each patient and subjected to both RDT and microscopic examination. Statistical analyses, including sensitivity, specificity, positive predictive value (PPV), negative predictive value (NPV), and overall diagnostic accuracy, were calculated using SPSS version 24.0. Multivariate logistic regression was conducted to adjust for potential confounders such as age, gender, and parasitic load.

Results

Out of 140 children, 83 (58.57%) tested positive for malaria by RDT, while microscopy confirmed 80 (57.14%) positive cases. The sensitivity of RDT was 96.25% (95% CI: 89.43%-99.22%), and the specificity was 90.00% (95% CI: 79.49%-96.24%). The PPV was 92.77%, and the NPV was 94.74%, with a diagnostic accuracy of 93.57%. Multivariate logistic regression analysis indicated that parasitic load was a significant predictor of RDT accuracy, while age and gender were not.

Conclusion

RDT is a highly accurate diagnostic tool for detecting *Plasmodium falciparum* malaria in children, demonstrating high sensitivity and specificity compared to microscopy. RDTs can be effectively used in resource-limited settings, but attention should be given to parasitemia levels to ensure accurate diagnosis.

## Introduction

Malaria is a life-threatening disease caused by parasites such as *Plasmodium falciparum* (Pf), *Plasmodium vivax* (Pv), *Plasmodium knowlesi* (Pk), *Plasmodium malariae* (Pm), and *Plasmodium ovale* (Po). Among these, Pf and Pv are the most prevalent and are associated with the highest mortality rates, particularly in endemic regions [1,2]. Despite being preventable and treatable, malaria continues to pose a major public health challenge, especially for children under five, who account for 61% of all malaria-related deaths globally. In 2017, approximately 219 million malaria cases were reported, resulting in nearly 435,000 deaths worldwide [3,4].

Children are particularly vulnerable to malaria, not only due to their underdeveloped immune systems but also because delayed or inaccurate diagnosis can lead to severe complications such as cerebral malaria, severe anemia, and even death [5]. The importance of early diagnosis in pediatric populations cannot be overstated, as prompt treatment significantly reduces the risk of mortality and other life-threatening complications. This vulnerability highlights the critical need for rapid and reliable diagnostic tools in resource-limited settings.

Traditionally, blood slide microscopy has been the gold standard for malaria diagnosis. However, this method is time-consuming, requires well-trained personnel, and depends on adequate laboratory infrastructure, which is often lacking in endemic areas, particularly in resource-limited regions [6]. Rapid diagnostic tests (RDTs) have been introduced as a more accessible alternative, offering faster results and easier application, especially in areas where laboratory capacity is limited.

RDTs work by detecting specific antigens produced by *Plasmodium* species in the blood. For example, histidine-rich protein 2 (HRP2) is specific to Pf, while aldolase and lactate dehydrogenase (LDH) can be detected in other *Plasmodium *species. This allows RDTs to differentiate between species, such as Pf-specific, Pv-specific, and pan-specific forms [7]. However, there are still concerns regarding the sensitivity and specificity of RDTs, particularly in cases of low parasitemia or mixed infections [8].

Given the limitations of microscopy and the variable performance of RDTs, especially in resource-limited settings, evaluating the diagnostic accuracy of RDTs in real-world conditions is crucial. This study was conducted in the endemic region of Peshawar, Pakistan, where malaria is highly prevalent among pediatric patients. Previous studies have reported variability in RDT performance, with some showing high sensitivity and others highlighting limitations in detecting low parasitic loads [9,10]. The current study aims to assess the diagnostic accuracy of RDTs in pediatric patients suspected of having malaria, using microscopy as the gold standard for comparison. By focusing on a vulnerable pediatric population, we hope to provide insights into the utility of RDTs in settings where access to reliable laboratory services is limited.

## Materials and methods

Study setting and participants

This retrospective cohort study was conducted in the Pediatric Department of Lady Reading Hospital Medical Teaching Institution (MTI), Peshawar, between January 1, 2023, and December 31, 2023. The study focused on pediatric patients aged 12 years or younger who were clinically suspected of malaria based on symptoms such as fever, chills, headache, and fatigue. Patients were excluded if they had a prior confirmed diagnosis of malaria before hospital admission, were already receiving anti-malarial treatment, or if their guardians did not provide consent for participation.

Intervention and diagnostic procedures

Upon admission, 3 mL of blood was collected from each child and subjected to two diagnostic methods: RDTs and microscopy. The RDT used in this study was the Paracheck-Pf kit (Orchid Biomedical Systems, Verna, India), which specifically detects Pf antigens in the blood. Thick and thin blood smears were prepared and examined by trained laboratory personnel. Thick film microscopy was used to assess parasitic load by counting the number of parasites per microliter of blood, which provides an estimate of infection severity. The parasite density was classified as low, moderate, or high based on the number of parasites detected per high-power field [5].

Blinding was not feasible due to the retrospective nature of the study, as the data were collected from existing medical records. However, trained laboratory personnel performed the microscopy analysis to ensure accurate diagnosis. Both diagnostic methods were conducted independently, and any discrepancies between RDT and microscopy results were recorded for further analysis.

Data collection and analysis

Data were extracted from patient records, including demographic variables (age, gender), clinical symptoms, and diagnostic results from both RDT and microscopy. The RDT results were recorded alongside the findings from microscopy, and discrepancies were noted.

Statistical Analysis

Statistical analysis was performed using SPSS version 24.0 (IBM Corp., Armonk, NY). Sensitivity, specificity, positive predictive value (PPV), negative predictive value (NPV), and overall diagnostic accuracy were calculated, with microscopy as the gold standard. Sensitivity was defined as the proportion of true positive cases detected by the RDT out of all cases confirmed by microscopy, while specificity was defined as the proportion of true negative cases among those confirmed negative by microscopy. Confidence intervals (CIs) for sensitivity and specificity were calculated to assess the precision of these estimates. A chi-square test was used to compare the diagnostic outcomes, with a p-value of less than 0.05 considered statistically significant.

To account for potential confounding variables, such as age, gender, and parasitic load, a multivariate logistic regression analysis was conducted. The parasitic load was included in the model as it significantly influences the sensitivity of RDTs, with higher parasitic loads increasing RDT sensitivity and lower loads leading to potential false negatives.

Ethical considerations

The study was conducted following the ethical standards set forth by the institutional review board of Lady Reading Hospital MTI, which granted approval for the research. Informed consent was obtained from the parents or guardians of all participants before data collection despite the retrospective nature of the study. All patient data were anonymized to protect confidentiality, and the ethical framework ensured that participants' rights and privacy were respected throughout the study.

## Results

A total of 140 pediatric patients were included in the study. The mean age of the patients was 6.8 ± 3.2 years. Of the total sample, 85 (60.71%) were male, and 55 (39.29%) were female. The age distribution showed that 62 (44.29%) patients were between zero and six years, while 78 (55.71%) were between seven and 12 years. The baseline demographics and clinical characteristics of the patients are presented in Table 1.

**Table 1:** Baseline characteristics of the study population This table displays the demographic and clinical characteristics of the 140 pediatric patients included in the study, categorized by age, gender, and symptoms.

Variable	Total (N = 140)	Male (N = 85)	Female (N = 55)
Age (mean ± SD, years)	6.8 ± 3.2	6.9 ± 3.1	6.6 ± 3.3
Age group (N, %)			
0-6 years	62 (44.29%)	35 (41.18%)	27 (49.09%)
7-12 years	78 (55.71%)	50 (58.82%)	28 (50.91%)
Fever (N, %)	140 (100%)	85 (100%)	55 (100%)
Chills (N, %)	112 (80%)	66 (77.65%)	46 (83.64%)
Headache (N, %)	87 (62.14%)	50 (58.82%)	37 (67.27%)
Fatigue (N, %)	93 (66.43%)	55 (64.71%)	38 (69.09%)

Among the 140 patients included, microscopy confirmed that 80 (57.14%) were positive for Pf malaria. The RDT identified 83 (58.57%) patients as positive. A comparison of RDT results with microscopy revealed 77 true positives, six false positives, 54 true negatives, and three false negatives.

The diagnostic accuracy of the RDT was evaluated using sensitivity, specificity, PPV, and NPV. The sensitivity of the RDT was 96.25%, while the specificity was 90.00%. The PPV and NPV were 92.77% and 94.74%, respectively, with an overall diagnostic accuracy of 93.57%. Table 2 summarizes the diagnostic performance of the RDT compared to microscopy.

**Table 2:** Diagnostic performance of RDT compared to microscopy This table outlines the sensitivity, specificity, PPV, NPV, and overall diagnostic accuracy of the RDT in comparison with microscopy, along with their 95% confidence intervals. NPV, negative predictive value; PPV, positive predictive value; RDT, rapid diagnostic test

Diagnostic parameter	Value (%)	95% confidence interval
Sensitivity	96.25%	89.43-99.22
Specificity	90.00%	79.49-96.24
PPV	92.77%	85.11-97.19
NPV	94.74%	85.81-98.82
Overall accuracy	93.57%	87.53-97.17

The sensitivity of the RDT was influenced by parasitic load. Patients with higher parasitic loads showed increased sensitivity. For patients with parasitic loads greater than 10,000 parasites/µL, the sensitivity of the RDT was 99.12%. In contrast, for patients with parasitic loads below 5,000 parasites/µL, the sensitivity dropped to 80.00%. The relationship between parasitic load and RDT sensitivity is detailed in Table 3.

**Table 3:** Sensitivity of RDT by parasitic load This table illustrates the sensitivity of the RDT based on parasitic load, with the corresponding number of false negatives in each parasitic load category. RDT, rapid diagnostic test

Parasitic load (parasites/µL)	Sensitivity (%)	False negatives (N, %)
>10,000	99.12%	0 (0%)
5,000-10,000	97.50%	1 (0.71%)
<5,000	80.00%	2 (1.43%)

Multivariate logistic regression was performed to evaluate the impact of age, gender, and parasitic load on the diagnostic performance of the RDT. Parasitic load was found to be a significant predictor of RDT sensitivity (p < 0.01), while age (p = 0.67) and gender (p = 0.81) were not statistically significant. Table 4 presents the odds ratios for the variables included in the logistic regression model.

**Table 4:** Multivariate logistic regression analysis This table presents the odds ratios and 95% confidence intervals for age, gender, and parasitic load in predicting the sensitivity of the rapid diagnostic test (RDT) based on a multivariate logistic regression model.

Variable	Odds ratio	95% confidence interval	p-value
Age (0-6 vs. 7-12)	1.08	0.72-1.61	0.67
Gender (male)	1.12	0.72-1.74	0.81
Parasitic load > 10,000	2.85	1.76-4.61	<0.01

**Figure 1:**
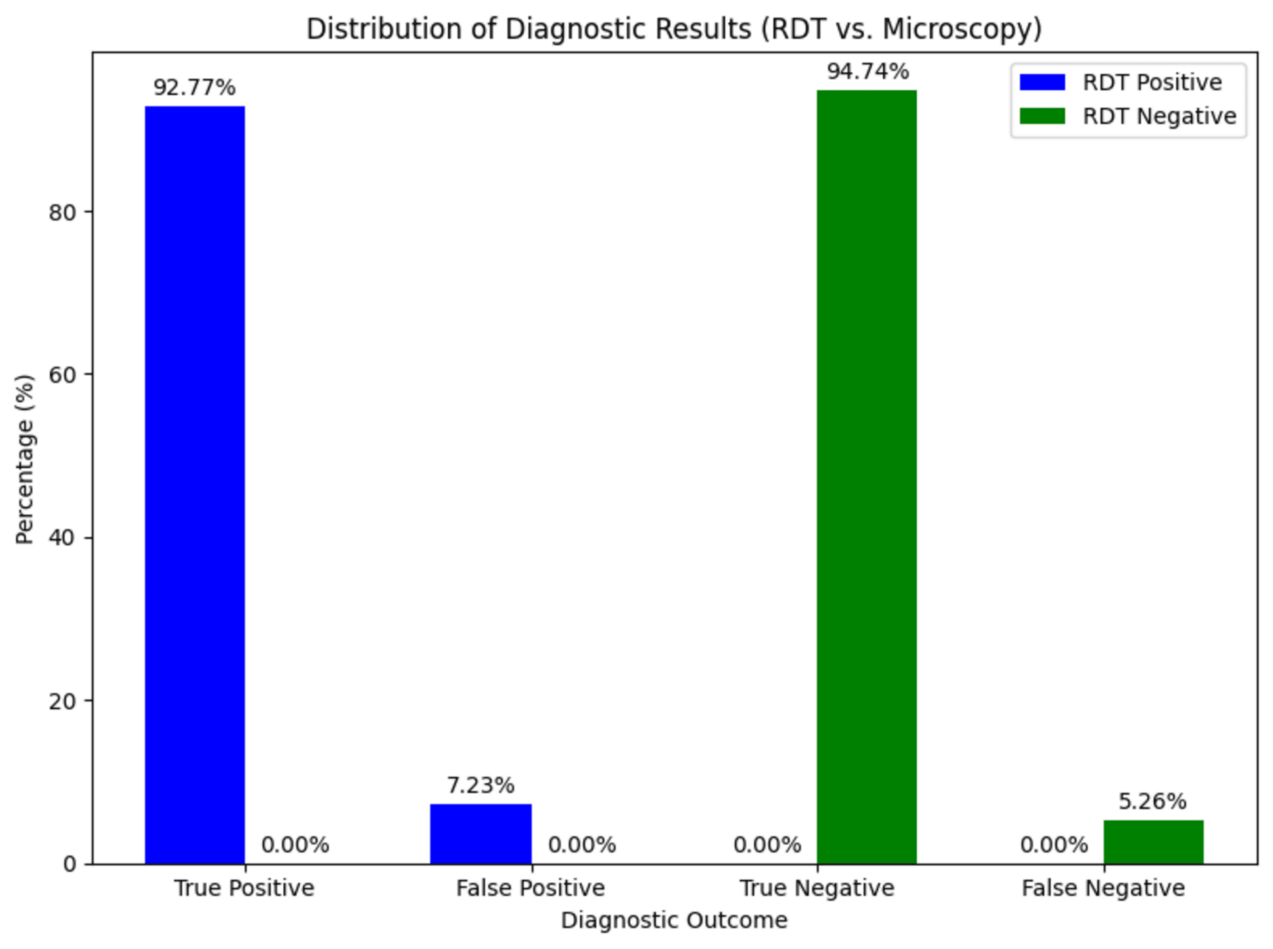
Distribution of diagnostic results (RDT vs. microscopy) RDT, rapid diagnostic test

The distribution of true positives, false positives, true negatives, and false negatives between RDT and microscopy is illustrated in Figure 1. The figure shows the proportions of each category, with the total positive and negative percentages set at 100%.

Figure 1 shows the distribution of diagnostic outcomes for RDT compared to microscopy. The total RDT positive results (n = 83) and RDT negative results (n = 57) are each divided into true positives/false positives and true negatives/false negatives, respectively. 

The RDT demonstrated high diagnostic accuracy, with sensitivity and specificity rates of 96.25% and 90.00%, respectively. However, parasitic load significantly influenced the sensitivity of the RDT, with lower parasitemia leading to false-negative results, particularly in patients with less than 5,000 parasites/µL.

## Discussion

Malaria remains one of the most common and serious infectious diseases globally, particularly affecting children in developing countries where resources are limited and public health infrastructure is often inadequate [11,12]. These challenges contribute to the persistently high prevalence of malaria in such regions. Accurate diagnosis plays a crucial role in reducing the complications and mortality associated with malaria. While various diagnostic methods are available, microscopy has long been considered the gold standard due to its ability to detect even low levels of parasitemia [13]. However, microscopy's reliance on specialized equipment and trained personnel limits its applicability in resource-poor settings. RDTs offer an accessible alternative, but their diagnostic accuracy, especially compared to microscopy, remains an area of interest.

In this study, 140 pediatric patients suspected of malaria were evaluated, with 85 (60.71%) males and 55 (39.29%) females enrolled. The majority of the patients (62 or 44.29%) were aged between zero and six years, which aligns with previous findings that show males and younger children are often more affected by malaria [14,15]. The high malaria prevalence of 57.14% (N = 80) in this cohort is higher than what has been reported in some other studies but can be attributed to the endemic nature of the region and the specific study period. For example, Acheampong et al. [16] reported that 18.6% of patients had confirmed malaria cases based on microscopy, while Khan et al. [17] reported that 1,381 (27.77%) out of 5,528 suspected malaria cases in Khyber Pakhtunkhwa, Pakistan, tested positive for malaria. These differences highlight the variability of malaria prevalence across regions and patient populations.

The results of this study revealed that while microscopy confirmed 80 (57.14%) cases of malaria, the RDT identified 83 (58.57%) as positive. The sensitivity, specificity, and diagnostic accuracy of the RDT were high, with values of 96.25%, 90.00%, and 93.57%, respectively. These results are consistent with the findings of Nkenfou et al., who reported lower, but still significant, diagnostic performance with an RDT [14]. The slight discrepancies between microscopy and RDT results, where the RDT identified three false negatives and six false positives, can be attributed to varying parasitic loads and possible cross-reactivity in the RDT used.

Other studies, such as those by Bisoffi et al. [18], have highlighted seasonal variability in RDT accuracy. Bisoffi's work demonstrated that sensitivity and specificity fluctuated between the dry and rainy seasons, with higher performance during the rainy season when malaria transmission is typically more intense. The influence of parasitemia is another critical factor, as shown in our study, where high parasitic loads (>10,000 parasites/µL) led to nearly perfect sensitivity (99.12%), while lower parasitic loads resulted in reduced sensitivity. This is in line with findings from Mahende et al. [19], who also reported high sensitivity but noted the performance of RDTs decreased with lower parasitemia levels. The variability in sensitivity, particularly at lower parasitic loads, points to the importance of considering parasitemia when interpreting RDT results in clinical practice.

Our findings also align with global studies from different regions, such as Iwuafor et al. [20], who reported lower sensitivity and specificity in their RDT evaluations. These differences could be due to population characteristics, environmental conditions, or the specific RDT brand used. The diagnostic performance of RDTs, while generally reliable, can fluctuate based on these factors, emphasizing the need for context-specific studies to inform local healthcare protocols.

Limitations

There are several limitations to this study that must be acknowledged. As a retrospective cohort study, our ability to control for confounding variables was limited, and reliance on medical records may have introduced selection bias. Additionally, the sample size, while adequate for this hospital, may not represent the broader pediatric population in regions with different malaria transmission dynamics. Seasonal variation in malaria incidence was not explicitly controlled, which could influence parasitemia levels and, by extension, the diagnostic performance of RDTs, as highlighted in other studies [16]. Another significant limitation is that the RDT used was specific to Pf, which may lead to underdiagnosis of other species like Pv.

Microscopy, while considered the gold standard, is subject to variability based on the skills of laboratory personnel and the level of parasitemia in patients. This could have led to an underestimation of false negatives. Future studies should aim to incorporate molecular diagnostic techniques, such as polymerase chain reaction (PCR), which can provide a more definitive diagnosis, especially in cases of low parasitemia or mixed-species infections.

## Conclusions

In conclusion, this study demonstrates that RDTs are a highly reliable method for diagnosing Pf malaria in pediatric patients, with sensitivity and specificity comparable to microscopy. However, the diagnostic accuracy of RDTs is influenced by factors such as parasitic load and the type of RDT used. Although false positives and false negatives were present, RDTs remain a valuable diagnostic tool in resource-limited settings where access to microscopy is constrained. Future research should focus on improving the specificity of RDTs, addressing the challenges associated with low parasitemia, and exploring multi-species RDTs to broaden the scope of malaria diagnosis.
